# Choice Hygiene for “Consumer Neuroscientists”? Ethical Considerations and Proposals for Future Endeavours

**DOI:** 10.3389/fnins.2021.612639

**Published:** 2022-06-13

**Authors:** Julia F. Christensen, Fahimeh Farahi, Meghedi Vartanian, Sina H. N. Yazdi

**Affiliations:** ^1^Department for Language and Literature, Max-Planck-Institute for Empirical Aesthetics, Frankfurt, Germany; ^2^3Fish Corporate Filmmaking, Tehran, Iran; ^3^Department of Psychology, University of Tehran, Tehran, Iran

**Keywords:** ethics, consumer neuroscience, neuromarketing, aesthetic emotions, addiction, obesity, moral dilemma, moral judgement

## Abstract

Is the use of psychological and neuroscientific methods for neuromarketing research always aligned with the principles of ethical research practice? Some neuromarketing endeavours have passed from informing consumers about available options, to helping to market as many products to consumers as possible. Needs are being engineered, using knowledge about the human brain to increase consumption further, regardless of individual, societal and environmental needs and capacities. In principle, the ground ethical principle of any scientist is to *further* individual, societal and environmental health and well-being with their work. If their findings can be used for the opposite, this must be part of the scientist’s considerations before engaging in such research and to make sure that the risks for misuse are minimised. Against this backdrop, we provide a series of real-life examples and a non-exhaustive literature review, to discuss in what way some practices in the neuromarketing domain may violate the Helsinki Declaration of Experimentation with Human Subjects. This declaration was set out to regulate biomedical research, but has since its inception been applied internationally also to behavioural and social research. We illustrate, point by point, how these ground ethical principles should be applied also to the neuromarketing domain. Indisputably, the growth in consumption is required due to current prevalent economical models. Thus, in the final part of the paper, we discuss how alternative models may be promotable to a larger public, aided by more ethical marketing endeavours, based on neuroscientific discoveries about the human brain. We propose this as a philosophical question, a point of discussion for the future, to make neuromarketing as a discipline, fit for the future, respecting the ethical implications of this research.

## Introduction

*“You can probably make them do anything for you: Sell people things they don’t need; make women who don’t know you fall in love with you.”*

– Vance Packard, *The Hidden Persuaders* (1957)

Consumer neuroscience is a young discipline. Concepts and methods from cognitive neuroscience are applied to research questions in the field of marketing. The aim is to develop a better understanding of consumer preferences and consumer behaviour. This endeavour has obvious potential benefits for individuals, businesses, society, and the environment. However, this marriage of science and the marketing industry is not (yet) a success story at all, due to the ethical implications of such research (Fisher et al., 2010; Stanton et al., 2017; Cherubino et al., 2019).

Our paper is specifically concerned with examples of misuse of methods and unethical research in the domain of consumer neuroscience. We are academics from other domains of research than neuromarketing. Therefore, our view should be understood as an outsider’s view on a domain of research that is still in the process of becoming, and where ethical concerns are still pending resolution which should be a concern for any researcher, outsider or not.

For the scope of this article, we will define neuromarketers as (neuro)scientists working in marketing departments in industry, or (neuro)scientists collaborating with industry partners on (neuro-) marketing questions from within a university (i.e., individuals with university education in psychology and/or cognitive neuroscience, working with businesses on marketing endeavours). Neuroscientists researching consumer choices without directly working with industry might also find our perspective useful. This article may also provide insights for classical marketers that seek to enhance their company’s marketing strategies with neuromarketing techniques, producers of ads, artists working in the marketing business, stakeholders in politics and policy-makers, and finally, for individuals/consumers of the societies of the future who seek information about the neurocognitive processes at play in our brains when we’re targetted by marketing strategies.

Within any academic and management field there is the possibility of unethical behaviour and conflicts of interest. The issues we flag in this article do not apply solely to the field of neuromarketing. Commendable initiatives like the International Neuroethics Society are concerned with such issues specifically within the realm of neuroscience. For the realm of neuromarketing, the Neuromarketing Science and Business Association (NMSBA) has provided a Code of Ethics, that members can sign up to voluntarily. These provide a useful starting point.

As in any field of research with human subjects, the neuromarketer has the responsibility to choose an ethical and sustainable research behaviour while still pursuing the strategic demands of stakeholders. If, on the contrary, no ethical code of conduct is observed, this is particularly problematic, as researchers are often based at universities, using publicly funded research facilities for their work with clients from the industry. In the words of Stanton et al. (2017):

“*Commercial Alert, a consumer advocacy group, sent a letter to the president of Emory University in 2003 alleging that neuromarketing is a significant risk to consumers and that Emory University should immediately halt all study of neuromarketing (Grey et al., 2003). In the letter, signed by academics and leaders of non-profit consumer advocacy groups, the authors state, Emory’s quest for a “buy button” in the human skull is an egregious violation of the very reason that a university exists. It also likely violates the principles of the Belmont Report, which sets out guidelines for research on human subjects in the United States. They go on to note, the real risk of neuromarketing research is to the people—including children—who are the real targets of this research. Already, marketing is deeply implicated in a host of pathologies. The nation is in the midst of an epidemic of marketing-related diseases.*”

Commercial Alert is an anti-advertising group that campaigns against many forms of marketing, not just against using neuroscientific methods in marketing endeavours. When Commercial Alert criticised neuromarketing at the beginning of the ’00, calls from promoters of neuromarketing were fast to dismiss potential dangers of neuromarketing to the individual. For example, Dr. Steven Quartz (a neuroscientist at the California Institute of Technology in Pasadena, California) was quoted for having said that such comments represented “gross misunderstandings and distortions of both the power of brain imaging technology and its use in marketing” (Blakeslee, 2004). Science has moved fast since then. While examples of misuse of neuroscientific methods for marketing purposes continue to be revealed, of course, it should be said that much of this research is conducted with adequate use of neuroscientific methods that help companies understand their consumers better.

US Department of Health and Human Services (1979) (mentioned in the quote from Stanton et al., 2017), along with several other codes of ethical research conduct (for an overview, e.g., Iphofen, 2009), all set out key ethical principles for any research endeavour involving human subjects. We will revise some of these proposed ethical principles in what follows in relation to neuromarketing.

For example, the Helsinki Declaration of Experimentation with Human Subjects (World Medical Association, 1964) sets out the ethical principles that regulate medical research. These principles are based in the The Universal Declaration of Human Rights (1948). Many international universities and research institutions that work in experimental psychology, cognitive neuroscience and data science (i.e., institutions where neuromarketers may work or may collaborate with) also adhere to these same principles. The European Commission (EURAXESS) (2000) holds that:

“*Research participants’ rights are anchored in fundamental human rights and the fundamental ethical principles that govern all scientific research. [*…*] Additional central policies and widely accepted declarations that codify principles of research ethics and ethical treatment of research participants include the Nuremberg Code, the Helsinki Declaration, and the Belmont Report. Although these codes originate in the biomedical field, they encompass the central principles that apply to all human research.”* (European Commission, 2018; pp. 4–5).

In what follows, by means of a non-exhaustive review of available literature in the field, together with real life examples, we illustrate how principles set out in these codes may be relevant in the context of neuromarketing. Neuromarketers must make a personal choice and bear their share of responsibility in what products they chose to help to promote, and how they do it. There is accumulating evidence that some advertising efforts, assisted by neuromarketing research, can have detrimental effects on the health of individuals, societies and the environment, potentially making more regulatory efforts necessary in the future.

Before we proceed review the ethical principles, some definitions of the object of study and a brief historical overview are in order.

### Marketing and Neuromarketing: Definitions and History

Marketing is the activity that seeks to shape and increase the sales of a product. In so doing, it seeks to create value for costumers and to capture value for the firm (Kotler and Keller, 2015). In marketing practice, there are four downstream tactics of marketing, or sub-domains of activities. Businesses develop marketing strategies for each of these four domains to ensure to market the right product to the right person, at the right price, in the right place, and at the right time (Cim, 2005). Detailed definitions from the perspective of the marketing discipline (both from the perspective of the academic study of marketing and of that of the marketplace practice of marketing) lie outside the scope of this paper. For such definitions, we recommend the work by Dolan (2019) and by Kotler and Keller (2015). We here provide short outsider insights before coming to the core of our objective with this paper, the ethical implications of some neuromarketing research.

Empirical research into people’s purchasing behaviour gained momentum in the beginning of the 20th century with Edward Bernays and Ernest Dichter (Bernays, 1928/1955/2004; Dichter, 1960/2012/2017; Papakonstantinou, 2019). Ever since, the aim of marketing research has been to shape sales through all four aforementioned domains. Today, neuromarketing methods are employed to target exactly the same four domains of sales-shaping (Dooley, 2011). See Table 1 for these four domains.

**TABLE 1:** The four Ps of marketing: product, price, place, and promotion (Kotler, 2003).

(1) What? The product. (i.e., anything that can be offered to a market from attention, to acquisition, to use or for consumption that might satisfy a want or a need (Kotler and Armstrong, 2010, p. 253).
Marketing activity: *Product design strategy* contents of the product, looks, taste, touch, sounds, wrappings, etc.
(2) How much? The price of the product. This is one of the most important elements of the marketing mix. If wisely chosen, it generates the turnover for the organisation (Low and Tan, 1995).
Marketing activity: *Pricing strategy* how much do people have to pay for it, etc. The price is a variable that should be set in relation to the other three Ps (Low and Tan, 1995).
(3) Where? The placement of the product (refers to how an organisation will distribute the product or service they are offering to the end user). Marketing activity: *Placement strategy* where will the product be offered (what shops, online/offline), where will the product be placed on the shelf, etc (Miller and Ginter, 1979).
(4) How? The promotion of the product (i.e., a vital part of business, it is an integral ingredient of the total marketing process).
Marketing activity: *Advertising strategy* decisions about design of ads, videos, online/offline, posters, etc. Promotion should make potential customers aware of the available products and services (Fam and Merrilees, 1998).

*Neuromarketing methods can be employed within each of these four sub-activities of marketing.*

Neuromarketing endeavours seek information and insights beyond those obtained by traditional techniques such as surveys, focus groups, and ethnography (Yoon et al., 2012; Plassmann and Weber, 2015), to improve the accuracy of predictions of consumer preferences and behaviour when combined with traditional techniques (Venkatraman et al., 2012; Smidts et al., 2014; Boksem and Smidts, 2015). In the next three sections, we will give a brief overview of the history of marketing endeavours and how they are shaping both present and future endeavours. We specifically focus on information about the advertising strategy, the 4th P (persuasion and communication), since reviewing all four Ps in detail would go beyond the scope of this paper.

### Neuromarketing *Past*

Although only gaining momentum in the past couple of decades, neuromarketing has its roots in the beginnings of marketing as a discipline. The presumed ethically problematic issues (that we will discuss in this article), arise from a shift that has happened in mainstream marketing objectives:

• From: “*informing people about available options*”

(informative and complementary marketing) …

• To: “*make people buy more than what they need to boost income*”

(persuasive marketing).

#### Where Marketing Came From

During the industrial revolution, marketing was an effort aimed at how to produce and distribute goods at the lowest possible cost and to inform people about available options. In the 20th century, the focus shifted because the market was increasingly more crowded by several producers offering similar products. Marketing became an effort aimed at persuading people that the goods of one producer were better than those of another. Today, the markets are saturated, and companies compete for customers like never before. Their effort is now to understand costumers’ potential needs and to persuade customers to purchase products to fulfil these needs.

#### Examples of “Helping Consumers Get What They ‘Need’ “

Edward Bernays, a cousin of Sigmund Freund revolutionised this field. Based on the principles of psychoanalysis, Bernays presented a new vision to companies: Consumers’ minds work according to unspoken (and even unconscious) feelings and desires “*that you cannot ask them about in an interview.*” Hence, the science of “unlocking the consumer’s unconscious mind” was born (Bernays, 1928/1955/2004).

One first landmark example of the techniques used for persuasion was the “Torches of Freedom.” Edwards Bernays was hired by a tobacco company to do something about the “problem” that women were not smoking (which caused the tobacco industry to “lose out” on possible revenues). This was in the 1920s, and it was considered shameful for women to smoke in public. However, overhearing conversations of potential cigarette consumers, he understood that feminists associated (at the time) smoking with freedom, and that, for them, the cigarettes were “torches of freedom.” Bernays informed newspapers that during the Easter parade of that year, a group of remarkable women would light “Torches of Freedom.” To capture this event, a large number of reporters attended the occasion and captured on camera how a group of very fashionable ladies lit their cigarettes in unison. This marked a new trend: “the modern independent woman, …*smokes*” – wonderful PR for the cigarette: *“If you smoke, you’re a free woman.”* The press photographs went “viral” in terms of then, and from then on, cigarette sales increased, and, although causality is always difficult to establish in a real life context, it should be noted that also the prevalence for lung-cancer in women increased (Grannis, 2017).

Similar manipulations of social norms and health behaviour were performed to boost the sales of a company by Ernest Dichter in the 1950s, when instant foods started to appear on the markets. Ernest Dichter was another psychoanalyst, and he invented the “focus groups” based on the free association group sessions of psychoanalysis (Dichter, 1960/2012/2017). Eves-dropping of the target groups as they tried out new products, he understood their joys and concerns alike, and also their dilemmas. This was how a simple egg made the sales of the brand Betty Crocher Foods skyrocket. *In theory*, housewives really wanted to ease their domestic tasks, so instant products were attractive for them. However, sales didn’t mirror this desire. Why? During focus groups, Dichter realised that, *in practice*, the housewives felt guilty for making their life easier by using instant products when cooking for their husbands. Although it wasn’t necessary, Dichter proposed the manufacturer make it compulsory to add a fresh egg to the mixture, to return some agency of the processes to the housewives’ hands, and to give them “a sense of participation” and it did the trick: sales increased dramatically. Apparently, having to add an egg to the mixture (even if that egg could, technically, have been part of the instant product already in the package) relieved the housewives of their guilt of using instant products. Sales of instant foods increased. Direct links between instant foods and obesity are still under investigation, however, based on first data, a link between high consumption of processed foods (including instant foods) and obesity is likely (Poti et al., 2017; Askari et al., 2020).

#### Is “Defence Breaking” Ethical?

Let us start by considering these two historical examples through the lens of the topic of this article: ethical research practices. Throughout the 20th century, experimental psychology and other behavioural/social sciences have seen dreadful examples of research misconduct that were only possible because no ethical codes were yet in place to regulate these endeavours. For example, the obedience experiments of Stanley Milgram or the Stanford Prison Experiments all had detrimental effects on research participants’ mental and physical health after their participation in these. Such examples raised awareness and highlighted the need to formulate and adhere to ethical research principles to safeguard research participants, not only in medical but also in behavioural research.

In both examples from Section “Examples of “Helping Consumers Get What They ‘Need’ “” above, the social emotions shame and guilt (Tangney et al., 2007) were “hindering” people from purchasing the manufacturers’ product (e.g., cigarettes, instant foods), and the marketing process used in both cases, removed peoples’ personal defences, “helping” people to smoke and to buy instant food products. From an ethical research practices point of view, let us consider two points:

First, ever since Aristotle, emotions like shame and guilt have been mentioned as important regulators of our social behaviours within society. Modern research from psychology and affective neuroscience has illustrated how the emotions of shame and guilt are evoked when we don’t act according to personal and societal values or rules. They are, thus, important regulators of our behaviour, also referred to as moral and aesthetic emotions (Tangney et al., 2007; Dempsey, 2017; Menninghaus et al., 2019). It may, therefore, be necessary to evaluate whether it is ethical to break down such natural “defences” of human cognition, effectively deceiving individuals into acting in discordance with their own values.

Second, considering what we know about human psychological and physical health *today*, neither cigarettes nor instant foods are healthy. In the beginnings of marketing as a discipline, there was little knowledge available about how smoking destroys the lungs, and about how the human brain reacts to addictive substances, and how habits and cravings develop in the brain. In the past, it was still not empirically established, how these processes stirred by marketing endeavours, boosting a business, could lead to wide-ranging negative effects for individuals’ mental and physical health, for societies, e.g., due to increasing costs to health systems; exploitation of the workforce, and for the environment, e.g., due to pollution or non-sustainable use of resources.

Today, evidence with regards to such detrimental effects is accumulating. Therefore, and importantly in this piece, research that contributes to such “defence breaking” wouldn’t be in accordance with ethical research practice as it is understood today.

### Neuromarketing *Present*

Current neuromarketing endeavours are based on the assumption that what one craves is also actually what one needs. This assumption is not aligned with current knowledge from affective neuroscience and psychology. Over-availability of opportunities for hedonism can corrupt our mental health and the choices we make in our life (Christensen, 2017).

In this section, we will review some ethical implications of present-day food and drink advertisement for sugary-products. Such advertisement can be one of the factors contributing to leading large segments of society into obesity-related health problems and is, therefore, an ethically-relevant issue.

The current situation is the following: Our urban visual and auditory environment is crowded with cues related to our basic and secondary needs (food, drink, sex, shelter, safety, status, etc; Maslow, 1954). Such cues remind us of hedonic experiences and, make us crave products that may give us these hedonic experiences. Thus, without any education about how our brain reacts to such cues, these can be detrimental for individual, societal and environmental health. Official health education about “pleasure” does not currently exist, nor do advertisements include disclaimers that inform consumers of these processes. Ethical research practice in neuromarketing for food advertisement must, therefore, include considerations about the possible consequences of manipulations of the human reward system can lead to detrimental effects for the individual, society and the environment.

**FIGURE 1:**
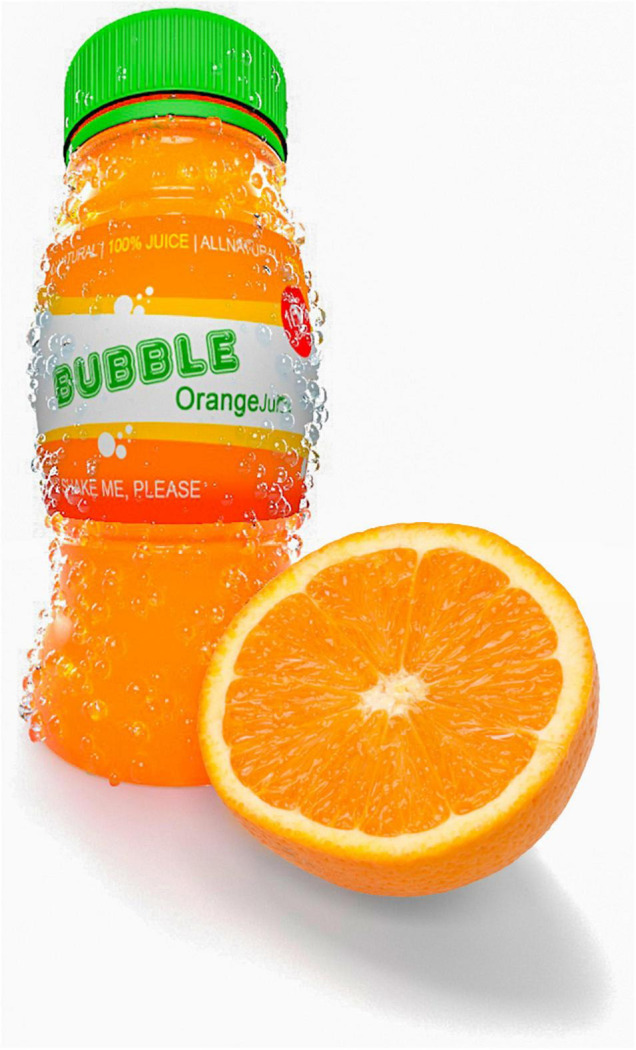
The Orange Bubble Juice from our Hypothetical Orange Bubble Juice Dilemma. Copyright: Sina HN Yazdi.

In what follows we attempt to outline the fine line between ethical and unethical experiments in the neuromarketing realm by means of The Orange Bubble Juice Ad Dilemma. The objective of this exaggerated and provocative example is to raise awareness of potential ethically relevant issues in neuromarketing. Box 1 and Figure 1 set out The Orange Bubble Juice Ad Dilemma.

**Box 1. Figure 1. Example.**

As a hypothetical dilemma that a neuromarketer may find themselves in, consider the following collaboration between marketers and neuroscientists for a new type of sparkling orange juice for children, The Orange Bubble Juice. Neuromarketing and experimental psychologists are invited as collaborators from a university to help to market this new juice in the following way, acting on all four domains of marketing outlined in Section “Marketing and Neuromarketing: Some Definitions and History”).

For the *product design strategy*, the neuromarketers proceed to design two types of experiments. Experiment 1 aims to determine which chemical composition of the juice pleases children the most. For instance, from research with other drinks, it is known that high levels of sugar are very pleasing due to the reward-related activations that sugar causes in the brain, and therefore induces the person to seek to drink more of the substance (and thus, boost sales). However, through negative taste bud-brain feedback loops, the brain will usually send signals to the gut that makes us feel satiated when unhealthy levels of sugar are reached. This stops the person from drinking more, which would be “unfortunate” for sales (i.e., sales would decrease). Let us assume that previous experiments have shown that adding the right amount of CO2 (bubbles) will numb the taste buds and facilitate ingestion of sugar, also beyond healthy levels. Experiment 1 will thus find the right ratio of sugar/bubbles (Di Salle et al., 2013; Sternini, 2013). Experiment 2 builds on the research that has shown that the artificial combination of a high number of different flavours that don’t exist in nature, induces strong hedonic feelings due to strong activation of the reward system of the brain. Thus, experiment 2 seeks to determine the right mix of artificial flavours that would make the Orange Bubble Juice irresistible (Flood-Obbagy and Rolls, 2009; Endrizzi et al., 2019).

For the *pricing strategy*, the neuromarketers help to find the right number combination that causes a perceptual bias in the consumer (e.g., 1.99€ instead of 2€; Sands and Sands, 2012), or deciding how to overcrowd the price tag with multiple items making it difficult to distinguish the real price of each piece (Dooley, 2011). Besides, there are additional cognitive biases in the economic domain that can be used to design the pricing strategy, such as the “*today only, two for the price of one*” -strategy.

For the *placement strategy*, research about overstimulation of the senses and ego depletion might illuminate the best placement option for the product. If we place the Orange Bubble Juice, for instance, close to the cashier, and the packaging design has been successful, children are likely to see the product while waiting with their parents at the cashier. This placement strategy might cause some temper tantrums and uncomfortable moments for the others in the queue. However, it is a useful strategy to increase sales because parents often succumb to the shameful feelings caused by the temper tantrum of their child: They buy the product just to avoid the uncomfortable situation. Several research papers analyse the behaviour of children in supermarkets and discuss it as a health-problem. For the purposes of boosting the sales of the Orange Bubble Juice, this confirms that the parents of the children in the target group are likely to succumb to the tantrum, as in e.g., O’Dougherty et al. (2006); Maubach et al. (2009), Carnell et al. (2011); Henry and Borzekowski (2011), Lesser et al. (2012); Swinburn et al. (2013), Winston et al. (2013); Haselhoff et al. (2014), Mason et al. (2014); Tipton (2014), and Rigo et al. (2018). For the placement strategy, in general, the diversity of product availability has broadened exponentially in the past decades, making choices effortful. Research shows that choices that use the body’s basic energy supply can, therefore, easily be depleted (Vohs et al., 2005), a process also referred to as ego-depletion. As a result, self-control fails and decision-making is impaired (Baumeister, 2002a,^2014^; Baumeister et al., 2008; Pocheptsova et al., 2009). Psychologists refer to this phenomenon as decision fatigue (Pignatiello et al., 2020), which may be an important cause of impulsive purchasing that can be followed by regret because of money spent (Baumeister, 2002b; Sharma et al., 2010). It can be exacerbated by environmental changes; for example, perceived crowding and also employee friendliness boost sales (Mattila and Wirtz, 2008), as does peer presence (Luo, 2005). The neuromarketers working on marketing the Orange Bubble Juice take all of these into account in the placement strategy, thanks to knowledge from neuroscientific and psychological research.

For the *promotion of the product*, the neuromarketers conduct experiments to find the best design of the ad, given the target group. This area of neuromarketing has expanded in the past years, with neuromarketers aiming their experiments at improving the ad design so that it stirs peoples’ emotions (Folkvord et al., 2016). The most efficient marketing efforts are those that tap into our basic needs. Maslow (1954) described different levels of needs of a person, the so-called basic needs (hunger, thirst, warmth, sex, rest), needs related to the person (safety needs, esteem needs, status, accomplishment, love, friends), and higher-order needs for self-realisation (achieving full potential, do creative activities, etc.). Whenever we fulfil one of these needs, we feel pleasure, and this makes us want to repeat (Kringelbach and Berridge, 2009, 2010a,b; Kringelbach et al., 2012). Much research has shown, that when needs are not filled, we crave the items, situations, or people that can help us quash the need (hunger, thirst, sex, etc; Maslow, 1964). Here, in our hypothetical example, depending on the target group of the orange juice, the neuromarketer could consider the socio-economic status of the parents and/or developmental stage of the child, and which needs might be at the forefront of the parent’s/the child’s brain, in order to design the ad to tap into that specific unfulfilled need “(real or engineered)” (Cao et al., 2013; Yunus et al., 2016). From a neuro-developmental point of view, children start to perceive the world around them around the age of 5 years and start appreciating the praise of their peers (Buss et al., 1979; Tangney et al., 2007; Dempsey, 2017). Until this time, advertisement related more to basic needs and perceptual stimulation is likely to be most successful. After that, ads related to person-needs like self-esteem and friends’ praise can be considered. Thus, for the promotion strategy of this hypothetical juice, experiments involve the assessment of which colours catch children’s attention most, whether the ad should include some “cool peers” of the target group, and whether or not, children react more to pictures of bottles where the configuration hints at a “hot summers day” (sweaty bottle and oranges in the pictures), like adults do, or not (see Folkvord et al., 2016, p. 27 and our Figure 1 for a short analysis). Let’s ask a provocative question: Would such endeavour be ethical? See Figure 1 for an illustration of the Hypothetical Orange Bubble Juice Dilemma.

#### Is Promoting a Sugary Juice With the Help of Neuromarketing Methods Ethical?

Of course, no university would approve an ethics application with the research objectives of the experiments outlined for our hypothetical Orange Bubble Juice example in Box 1. The obvious psychological and physical health hazards that they pose to the individual, children in this case (e.g., obesity), to society (e.g., increased cost to health services due to obesity), and to the environment (e.g., pollution through the surplus of plastic bottles) would make such research unethical from a university ethics board point of view. However, the hypothetical Orange Bubble Juice dilemma illustrates the trade-off that neuromarketers may at some point have to consider, between safeguarding individuals, society, and the environment on the one side, and of safeguarding and helping the broader economy, on the other. Therefore, let us see a brief overview of the evidence that we possess today about health hazards that the hypothetical Orange Bubble Juice would pose in these three domains.

Regarding *individual* health, the product’s chemical composition (product design) induces children to ingest more sugar than what is healthy (Harris et al., 2015). As is well-known, the building blocks of adult obesity are set in childhood (Longacre et al., 2016; Hartmann et al., 2017; Rigo et al., 2018), and dental health is among the most painful and immediate consequences of surplus sugar ingestion (Duijster et al., 2015; Mela and Woolner, 2018).

Regarding the *social* consequences, both obesity and dental destruction are high costs for the health services of societies that are paid by the taxpayer. Notably, for every $1 that the World Health Organisation spends on promoting nutrition, the food industry spends $ 500 on promoting processed foods (Boyland and Halford, 2011; Wold Health Organization, 2012–2013). Besides, on a small societal scale, the tantrums at the supermarket due to the product placement are clearly antisocial for the people around and yield high levels of stress in the parent. Other societal problems that have been related to the use of brain-hack methods from the marketing realm are social exclusion, negative social comparison processes, and exploitation of the workforce in third-world countries.

Regarding *environmental* consequences, to keep costs low, the production of chemical compounds and the wrapping is often outsourced to countries where worker’s rights and production laws are less stringent. This is a common societal problem of today’s world (Radfar et al., 2018), but also an environmental one since it leads to pollution, e.g., in terms of miles and in terms of pollution in the country of production where environmental laws may be less strict.

The Orange Bubble Juice Ad Dilemma illustrates that the way neuroscientific methods would be used to advertise this Orange Bubble Juice would not be aligned with current understandings of how to safeguard and promote human health and cognitive functioning. Such research would, therefore, not live up to current standards of what is considered ethical research practice for the social and behavioural sciences.

## The Helsinki Declaration of Experimentation With Human Subjects

The Helsinki Declaration of Experimentation with Human Subjects (HD) Association (1964) by the World Health Organization (WHO) sets out the ethical principles that regulate medical research, and that are based in the The Universal Declaration of Human Rights (1948). As underscored by several international directives (US Department of Health and Human Services, 1979; Iphofen, 2009; European Commission, 2018), these apply as much to medical research, as to social and behavioural research endeavours, to ensure the The Universal Declaration of Human Rights (1948) of research participants are respected and protected.

The HD was set out after the atrocities committed by “scientists” during World War 2, to avoid that, ever again, humans and animals be abused for the purposes of “science.” However, some may feel uncomfortable with the fact that we are now in a situation where it has become standard practice within some segments of research, to “use” human participants to understand how the human brain works with regard to purchasing decisions, with the objective to boost “the economy,” regardless of what negative effects there may be for us as *individuals* (McDevitt et al., 2000; Köster, 2009), our *society*, and our *environment*. For instance, the addictive nature of some devices for the human brain can result in over consumption (Duke and Montag, 2017b; van Velthoven et al., 2018; Noë et al., 2019; Yu and Sussman, 2020), and resulting e-waste can harm the environment (Needhidasan et al., 2014; Duke and Montag, 2017a; Joon et al., 2017; van Velthoven et al., 2018; Noë et al., 2019; Singh et al., 2020; Yu and Sussman, 2020).

The ground ethical principle of the HD is that any scientist should work to *further* individual, societal and environmental health and well-being with their work. If their findings can be used, explicitly or implicitly, for the opposite, this fact must be part of the neuromarketers’ ethical considerations and risk assessment before engaging in such research. In principle, researchers are committed to minimising risks for misuse. Besides, the HD is intended to be universal and binds researchers to an ethical creed, no matter where in the world they exercise their activity. Point 9 of the HD specifically stipulates that “*No national ethical, legal or regulatory requirement should be allowed to reduce or eliminate any of the protections for human subjects set forth in this Declaration.*” Even if a neuromarketer will not be involved specifically in researching the production in a different country, this doesn’t alleviate them of their responsibility to understand and question the production context of the product before agreeing to collaborate in marketing a product.

We now discuss four aspects that researchers in neuromarketing should consider carefully before helping any entity seeking a consumer neuroscience service. These four include (i) the use of deception, (ii) individual’s dignity, (iii) adequate use of methods, and (iv) effects on the environment of the (neuro)marketing effort.

### Deception

Point 20 of the HD stipulates that “*The subjects must be volunteers and informed participants in the research project*.” This point relates to the use of “deception” in research. Participants must be *informed*, and provide *informed consent*, if scientists are involved in the research.

An important cornerstone in the ethical considerations of any researcher undertaking experimentation is the decision about whether or not deception is part of the research protocol (Kim, 2012; Nijhawan et al., 2013); for discussions about this issue, see Miller and Kaptchuk (2008); Boynton et al. (2013), and Plunk and Grucza (2013). There can be research questions, where deception must be used for the results of an experiment to be useful (e.g., Bortolotti and Mameli, 2006). In such cases, however, the ethical application that will be revised by the university ethical board must include an important part that justifies this, a risk assessment, and importantly, a debrief sheet would be needed that informs the individual that they are being deceived.

An example of a blatant violation of the informed consent principle, illicit use of deception and absence of a debrief sheet, was a case where Facebook employees collaborated with academic researchers to conduct a study that intentionally manipulated nearly 700,000 users’ mood states without users’ consent (Kramer et al., 2014). The company received significant public backlash for not acquiring users’ informed consent in advance of participating in the study (Flick, 2016).

The HD requires the experimenter to inform the research participants of the objectives of the research, and to disclose that the knowledge gathered with the evidence from their participation in this research could potentially be used to market this product in the real world and thus produce the cited negative health outcomes to those that consume the product. Point 22 of the HD stipulates: “*In any research on human beings, each potential subject must be adequately informed of the aims, methods, sources of funding, any possible conflicts of interest, institutional affiliations of the researcher, the anticipated benefits and potential risks of the study and the discomfort it may entail*.” And any research, including neuromarketing research, must respect point 19 that *“[*…*] research is only justified if there is a reasonable likelihood that the populations in which the research is carried out stand to benefit from the results of the research.”* The neuromarketer should ideally evaluate their contribution to a marketing effort taking into account these considerations; what is the objective of the research and are the end-users likely to benefit from this effort, or eventually be disadvantaged?

The hypothetical Orange Bubble Juice dilemma above, just as the examples of the Torches of Freedom and the “added egg” of Betty Crocher Foods instant foods, all include methods aimed at deceiving individuals into doing, thinking or feeling something that they wouldn’t otherwise be doing, thinking or feeling. Brief, the strategies used (informed by psychology and neuroscientific evidence about the human brain) exploit the functioning of peoples’ brain to make them consume something they otherwise wouldn’t. Of course, *false* advertising is a crime, however, the boundaries between what is true and false are often blurry, which makes such laws difficult to enforce. Even so, the US Federal Trade Commission (FTC) successfully sued Lumos Labs for their misleading advertising where they claimed that their “brain training” games could prevent Alzheimer’s Disease (Hufford, 2016).

If we continue with the hypothetical Bubble Orange Juice dilemma, the strategies used - hypothetically - could be seen as deceptive:

- Product design (combination of flavours, use of carbonisation, etc.) to induce individuals to ingest too much sugar (outcome for consumer: obesity, health complications);
- Juice placement close to cashier to manipulate child into having a temper tantrum (outcome for consumer: upsetting parents and the micro-society in a supermarket with temper tantrums, harming the parent-child relationship);
- Tricks on parents to make them buy the juice through ego depletion strategies or perceptual biases regarding the pricing (outcome for consumer: making individuals act in dissonance with their own values and convictions).

As stipulated by point 14 of the HD: “*The research protocol should always contain a statement of the ethical considerations involved and should indicate that there is compliance with the principles enunciated in this Declaration.*” Thus, if this was an experiment in a psychology lab-situation, given the possibility of these negative psychological and physiological health outcomes, the experimenter would be required to debrief the participant after the session: “We have produced the juice in such a way that you couldn’t stop drinking it, and a possible side-effect that we anticipated was a conflict between you and your parents.” This obviously sounds rather absurd in the context of a consumerist society, however, it illustrates the ethical principles against deception that researchers may want to abide by, within or outside academia.

To illustrate this point further, in the real-life context, no researcher is standing at the exit of any shop to “debrief” consumers, telling people that their purchases were engineered, and likely biased by, e.g., perceptual biases in number perception. However, if an ethical code of conduct is to be respected, then presenting an ad that involves elements of deception, does not stop being deceptive. Besides, theoretically, wouldn’t this be against the human right of self-determination (The Universal Declaration of Human Rights, 1948, article 1, 2 and chapter IX, article 55), when the same stimulus (ad) is presented in a real-life context? Conceded, this may again be a somewhat philosophical question and such an idea may seem difficult to implement in the real-life context. Nevertheless, it may still be a question we want to think about as a society.

**FIGURE 2:**
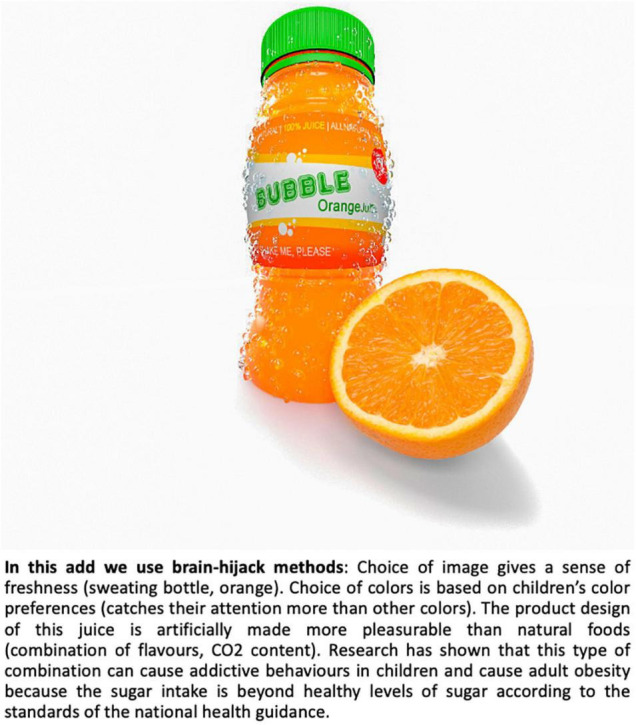
Hypothetical debriefing, or “information signalling” on the ad of the Orange Bubble Juice of our Hypothetical Orange Bubble Juice Dilemma. This “debriefing text” is intended to be thought provoking, and maybe somewhat provocative, as is the whole thought experiment about the Hypothetical Orange Bubble Juice Dilemma. Copyright: Sina HN Yazdi.

Such “debriefing” efforts, disclaimers, or other “information signalling” are starting to emerge in some countries. For example, the European Tobacco Products Directive (2014/40/EU) imposed in 2016 that health hazards derived from smoking must be printed on cigarette packages. Examples of other initiatives include nutrition labels with a colour code indicating overall nutritional quality (e.g., red, yellow or green) (Ducrot et al., 2016), or signalling with stickers that give information about nutrient contents at a glance (Egnell et al., 2018). Awareness of the meaning of these signalling etiquettes is not always high among the most vulnerable groups such as children and adolescents (Wojcicki and Heyman, 2012a). However, such initiatives, *at least*, have the potential to inform -or, debrief- the user. If such “disclaimers” were implemented more widely, it would be in keeping with international codes of research conduct: If neuromarketing methods have been used with manipulative intent (e.g., ad and product have been designed so that they likely reduce consumers natural self-restraint; the pricing and placement of the product follow strategies from cognitive science that result in perceptual biases that cause ego depletion and, eventually, impulsive buying in the consumer, etc.), the consumer must be informed about this at purchase. Promotional ads could include a short clause, about the type of deception used in the ad. For an arguably provocative example of a disclaimer for an ad, see the lower part of Figure 2.

### Dignity

In 1957, Vance Packard published his seminal book *The Hidden Persuaders*, and one quote from the book goes as follows: *“At one of the largest advertising agencies in America psychologists on the staff are probing sample humans in an attempt to find how to identify, and beam messages to, people of high anxiety, body consciousness, hostility, passiveness, and so on”* (Packard, 1957/2007). This quote expresses a moral outrage at a company having the objective to manipulate us into buying something we don’t need, based on our personality, moods, or other characteristics. It seems incredibly naïve seen from the perspective of today. It is now a mainstream marketing effort to use personality profiles and other “psychographic” variables of the target groups (e.g., Kotler, 1965; Henriques et al., 2009; Ghosh, 2010; Cisek et al., 2014; Cai et al., 2015; Graffeo et al., 2015; Udomkun et al., 2018; Ardeshiri et al., 2019). It became even more mainstream with the launch of Google personalised ads in 2005.

With the help of psychologists and neuroscientists, marketing personnel has long realised that “*past behaviour, habit and hedonic appreciation are usually better predictors of actual food choice behaviour than psychological constructs like attitudes and intentions”* (Köster, 2009). Most of our choices are not based on rational and reasoned deliberation, and there is often an intention-behaviour gap that can be exploited to boost sales, and which is mostly grounded in personality traits and life style (Weijzen et al., 2009). These can be targetted (/exploited) quite efficiently through personalised ads.

Let us see a couple of examples. One prominent neuromarketing firm proposes the possibility to use Smart TVs in the future to provide personalised ads to TV audiences. According to their figures, the use of smart TVs increased by 12% in the past years, and they see this as a great opportunity for advertisers to target people with personalized ads while they watch TV.

#### Is This Ethical?

If considering point 21 of the HD, which is reminiscent of article 1 of the Charter of Human Rights (“*All human beings are born free and equal in dignity and rights”*), and of article 22 of the same Charter (which affirms each individual’s economic, social and cultural rights that are indispensable for their dignity and the free development of their personality), we may question whether the above objective with Smart TVs would be considered ethical from a research ethics perspective. Point 21 of the HD posits that “*The right of research subjects to safeguard their integrity must always be respected. Every precaution should be taken to respect the privacy of the subject, the confidentiality of the patient’s information and to minimise the impact of the study on the subject’s physical and mental integrity and on the personality of the subject*.” Note especially the latter part of this point. The impact of a marketing endeavour for such Smart TV, with successful product design, pricing strategy, and placement strategy, does *not* preserve the individuals’ physical and mental integrity and personality, if it is aimed at conditioning our behaviour to be in a way that it wouldn’t naturally occur. To cite just one example that would speak against the use of Smart TVs for personalized advertisement is that TV viewing is already heavily associated with snaking behaviour, that is, children and adults ingest quantities of unhealthy foods they otherwise wouldn’t have eaten (Thomson et al., 2008; Kelly et al., 2010; Parvanta et al., 2010; Thorp et al., 2013). If university-based neuroscientific tools and evidence gathered with these contribute to individuals in some way losing control over their own actions, experience cognitive dissonance, feel upset, etc., this is in breach of the principles of ethical research. Researchers must *“[*…*] protect the life, health, privacy, and dignity of the human subject”* (HD, point 10), and make sure that *“[*…*] considerations related to the well-being of the human subject [*…*] take precedence over the interests of science and society.”* (HD, point 5).

As another example, let us consider smokers who have a dependence on cigarettes. Previous research has shown that their drug dependence can be tracked with neuroimaging techniques (McClernon, 2009). Based on this knowledge, and with the help of neuromarketers in product development, cigarette manufacturers can now test groups of cigarette addicts’ physiological and/or brain responses when they are presented with new varieties of cigarettes. This could help find out which designs engage brain systems associated with reward and reinforcement best, with the aim to choose those with higher addictive potential (Bates and Rowell, 2004). Wouldn’t such practices be questionable ethically, for example, due to the links that we know of today, between cigarette smoking and cancer (e.g., Stanton et al., 2016), and between addictive behaviour and other negative health consequences? It is known today that addiction impairs healthy decision-making and promoting substances that may lead to addiction does not seem to be in accordance with safeguarding individuals’ dignity and free development of personality.

Let us now consider several additional empirical examples as an overview, about how multisensory stimulation through the product and promotion strategy is used to target senses of consumers in an unprecedented way. There is of course, in principle, nothing wrong with accumulating knowledge about consumer behaviour. However, the education of society should go hand in hand with any such endaveours.

*Vision*. In the food industry, it is commonly suggested that, for unhealthy foods, ads stimulating multisensory channels “work best” (Elder and Krishna, 2010), while single-sense ads are successful to advertise healthy foods (Roose and Mulier, 2020), and that manipulation of the visual field (e.g., background/packaging/colour, dark/pale) can lead to differences in expected flavour and boost sales indirectly (Carvalho et al., 2017; Spence, 2019). Colour, shape, size, and shining transparency, reflections, and special textures can play a role in costumers’ decision-making processes (Manenti, 2013). In the beauty industry, it has been shown that among alternative products having the same function and price, those that are visually more appealing are more likely to be chosen (Creusen and Schoormans, 2005; Kim, 2010), and the visual product aesthetics is used to attract the consumer (Workman and Caldwell, 2007). For example, 40 percent of all perfume purchase decisions are based on the design of the bottle (Lindstrom, 2008).

*Audition*. Sound manipulation includes, for example, being exposed to positive music while tasting a product (Zampini and Spence, 2012). Research has shown that, for example, music can make a beer taste more appealing and sweeter to consumers, (North, 2012; Hauck and Hecht, 2019; Reinoso-Carvalho et al., 2019). Likewise, sounds related to the packaging or pouring of the liquid, and even the sounds of carbonation of a drink in a glass influence consumer’s multisensory tasting experience (Spence and Wang, 2015; Wang and Spence, 2019). In one study, participants rated their coffee taste differently when hearing the sound of a coffee maker machine versus when there was no sound (Knöferle, 2012), and ate more potato chips if the packaging was proportionate to its content’s crunchy nature (Smith, 2011). The sound manipulation can also be the sound of an aerosol spray (Spence and Zampini, 2007), or the background music playing in-store. Music is also used to create a brand identity, which can evoke a sense of pleasure, familiarity, and a willingness to spend more money and time. Classical music used to be played in Victoria’s Secret stores which created an atmosphere filled with prestige (Lindstrom, 2008). Many brands including New Look, Zara (Manenti, 2013), Hollister, and Abercrombie & Fitch have their specific playlist (Clarke et al., 2012).

*Smell*. Smell has been shown to be an effective means of connecting people with a specific brand. For example, Women’s Wear Daily reported in 2009 that Abercrombie & Fitch “ha[d] spent more than $3 million in the last two years on fragrance machines in its more than 350 stores” (Seckler et al., 2009). Several studies show that a pleasant fragrance positively influenced consumers’ affective reactions, evaluations, and intentions to revisit the store (Davies et al., 2003; Bosmans, 2006; Doucé and Janssens, 2013).

*Touch*. Another way to attract customers to a fashion product and make it more appealing is through touch (Spence and Gallace, 2011). It’s the first tool for apparel evaluation named ‘tactile marketing’ (Grohmann et al., 2007). It has four main characteristics: texture, hardness, temperature, and weight (Peck and Childers, 2003). In fact, the absence of tactile experience may reduce the chance of pleasurable shopping experience (e.g., online, TV). To overcome this pitfall, researchers have used sensory-enabling presentations, specifically, image zooming and rotation videos, and have measured cognitive and emotional reactions during product evaluation and purchase decision processes (Jai et al., 2014). These techniques are said to compensate for the lack of touch.

The ethical considerations for a neuromarketer approached to collaborate in experiments as in the examples above, is to determine in which cases such manipulation through the senses allows individuals to preserve their dignity and free development of their personality. Public outreach activities spearheaded by scientists from these domains may also contribute to spread the knowledge about these processes to society. This would allow individuals to make informed choices about which products to engage with.

### Ethical Use of Methods

Examples continue to surface indicating that that much research in the field of neuromarketing is unfortunately based on shaky assumptions about the human mind and brain, and neuroscientific research tools are applied without sufficient knowledge and training about their correct use, and importantly, about their limitations (see Stanton et al., 2017, for a review of such examples). It is the responsibility of the neuromarketer collaborating with firms from within universities, to manage expectations about the methods and their ability to generate meaningful insights for firms. Neuroscientific research might sound very “sexy” to some. However, if methods are not applied soundly, results are, unfortunately, deprived of any scientific validity. Besides, using human participants for research that is not underlying any validated scientific methods for the question asked, is also unethical.

For instance, the HD stipulates (in point 6), that “*even the best proven interventions must be evaluated continually through research for their safety, effectiveness, efficiency, accessibility and quality*”, (emphasis added by the authors). By implication, research that is not useful (i.e., without effectiveness) and not done well, i.e., done using incorrect methods (i.e., no efficiency), is unethical, as it doesn’t respond to the latest quality standards of psychological or neuroscientific research. The code, furthermore, stipulates that research should be “*conducted only by individuals with the appropriate ethics and scientific education, training and qualifications*” (point 12), and “*research involving human subjects must conform to generally accepted scientific principles, be based on a thorough knowledge of the scientific literature, other relevant sources of information*, (…)” (point 21). Stanton et al. (2017) who we quoted in Section “Introduction” with their serious qualms about consumer neuroscience, remind us of

“*The canonical criticisms of neuromarketing—which arose at its inception and have remained prevalent today— and which include unethical research practices, unethical applications of technology, and manipulations of consumers. Yet, despite these criticisms, the volume of academic research in neuromarketing and related areas has grown steadily and now over 200 neuromarketing research and consulting firms have been founded across the globe (Plassmann et al., 2012). With the growth of the field, criticisms and fears of neuromarketing’s purported power have not yet subsided— if anything they have grown.*”

Several well-known neuromarketing firms advertise online that they are able to use psychophysiological recording methodologies to give insights about “emotions” that consumers feel while watching an ad. They openly advertise that they are able to measure neurological and biological reactions of potential consumers and tie those to the success of an ad campaign, “lifting” sales significantly.

To mention just one review from the academic literature of carefully controlled research, we recommend the paper by Professor Kreibig. Her results show that there is no specificity of affective responses to be deducted from psychophysiological reactions (e.g., heart rate, sweat, posture, facial reactions, etc.) related to any categorical emotions (Kreibig, 2010). One can say, at best, that a psychophysiological reaction in response to a particular stimulus (if that has been time-locked within the experimental paradigm), correlates with a particular heightening or lowering in physiological response. Establishing causality is an entirely different question and requires careful experimental design.

Stanton et al. (2017), furthermore, argue that researchers in academia and neuromarketing have very different goals and approaches to collect the data and then interpret the result:

“(1) *Scientific results are worthwhile only if the methods used to collect the data are sound. Yet, industry clients who hire neuromarketing firms are not likely to have sufficient background knowledge to evaluate the methods used to collect and analyse neuroscientific data.*
(2) *Neuromarketing firms are incentivized to exaggerate their capabilities and potential deliverables to attract clients. Unlike the academic world, neuromarketing firms lack peer review when they report results to clients, and peer review protects against the risk of overstating results. For instance, the “case reports” that are often included on the web pages of such companies, are more often than not “internal reports” that have not passed by any quality filter, such as peer review.*
(3) *Moreover, neuromarketing firms tend to maintain proprietary control of data they collect. Neuromarketing firms also do not tend to publish or share their data collection protocols. This opacity means that the extent to which neuromarketing companies’ data are valid, or in correspondence with their promotional claims, remains unclear. In contrast, academic science utilizes peer review as a self-correcting feature.*”

Stanton and colleagues conclude that due to the sophistication and lack of tractability of neuromarketing research methods, compared to traditional marketing research, third-party evaluation agents, such as the Advertising Research Foundation (ARF), could be organised with the goal of delivering a quality certification. This would allow consumers of neuromarketing research to make a more informed choice regarding the product that they are purchasing from neuromarketing companies. See Venkatraman et al. (2015) for an example of such endeavour.

Another problem for companies interested in purchasing a neuromarketing service, is that when used properly, neuroscientific methods often do not show anything that the marketer didn’t already know (Harrison, 2008), which doesn’t justify the high costs of using neuroscientific methods.

**FIGURE 3:**
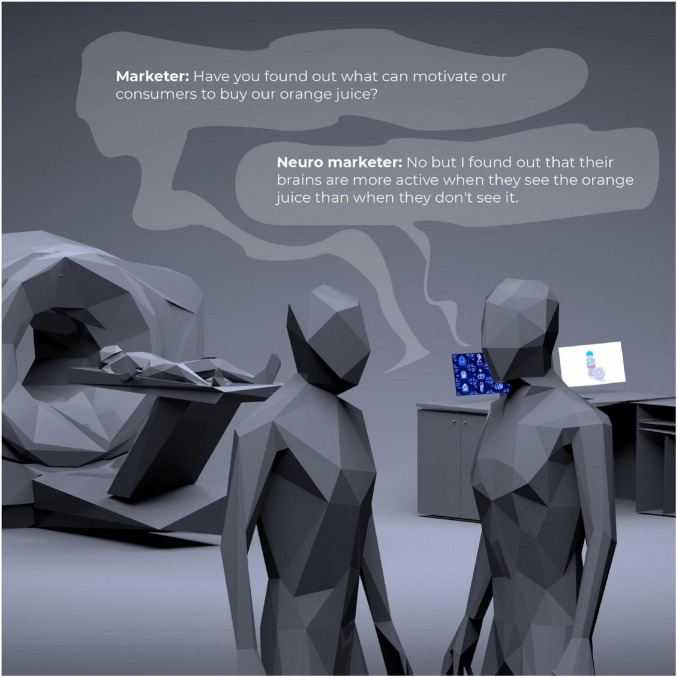
Illustration inspired by a well-known joke in neuromarketing. Copyright of the illustration within this paper: Sina HN Yazdi.

See Figure 3 for an example of an old joke regarding the power of neuromarketing to tell marketers anything that they didn’t already know.

### Environment

When research is conducted in collaboration with academic staff from publicly funded universities, universities require researchers to give some prior consideration to the potential misuse of research results, and they are asked to write a risk assessment. This includes potential harm or risks to individuals, societies, and the environment. In the case of neuromarketing research, this may include considerations about how to safeguard the latter three from these potential harms and risks. For this, three points of the HD are noteworthy: “*It is the duty of the [researcher] to promote and safeguard the health, well-being, and rights of [individuals], including those who are involved in [the] research. The [researcher’s] knowledge and conscience are dedicated to the fulfilment of this duty”* (point 4), and “*research should be conducted in a manner that minimises possible harm to the environment”* (point 11), and “*appropriate caution must be exercised in the conduct of research which may affect the environment, and the welfare of animals used for research must be respected”* (point 12).

Societies are increasingly consumerist, and this has a negative impact on individuals, societies, and the environment (Seiffert and Loch, 2005; Ghosh et al., 2016; Aschemann-Witzel et al., 2017; Rohm et al., 2017; Janssens et al., 2019). Several international bodies and associations have highlighted that we need a sustainable consumption strategy (Ellen, 1994). At the World Economic Forum (2012) it was emphasised that businesses might need support to reshape demand to promote sustainable consumption (Kaufmann and Panni, 2017). Neuroscientific knowledge about behavioural change can be useful to help businesses change the “fast consumption” mindset to a “sustainable consumption” mindset, such as, for instance, the promotion of a circular economic model (Milios, 2018; Ruiz-Real et al., 2018; Moraga et al., 2019), or emphasise the possibility of developing sharing economies (Querbes, 2018; Sands et al., 2020). However, if neuromarketers collaborate for the opposite to help increase consumerist behaviour, this should potentially be considered as violating the HD in terms of working to safeguard our environment.

## Future Neuromarketing?

One argument put forward by companies using persuasive advertisement is that we are all free to choose to purchase the products, services, experiences, etc., that they promote, or not. The companies do not oblige or coerce anyone into consuming.

And, that’s the crux of the problem:

Are we free, and do we choose what we *do* and *don’t do* at our own *free will*? Am I the *true* agent of my actions?

The Free Will debate has a long tradition within philosophy. The view that we have a “Free Will” (Dennett, 2003, 2004) is counterposed to deterministic views (Caruso, 2012, 2018). The deterministic, or, consequentialist view contends that our present behaviour is conditioned by previous events, and thus our actions are not entirely *free*, they are pre-*determined*. Events that happened in the past *cause* us to act in a particular way in the present. Notably, this view has received some backing from research in cognitive neuroscience. One seminal study by Libet and colleagues showed that research participants’ brain activity suggested that there was an activation pattern of action preparation, measurable in the motor cortices of the brain, milliseconds *before* the individual *was aware* of wanting to perform that action (Libet et al., 1983). This finding has been confirmed repeatedly ever since, and it implies that we are not entirely the conscious agents of our actions, as we like to think (Haggard et al., 2002; Engbert et al., 2008; Filevich et al., 2013).

In Section “Dignity,” we outlined examples of how specific variations in perceptual features (vision, audition, touch, smell, etc.) increased purchasing behaviour. Were these consumers entirely “free” when choosing? If it is true that “*past behaviour, habit, and hedonic appreciation are better predictors of actual food choice behaviour than psychological constructs like attitudes and intentions”* (Köster, 2009), then it would be recommendable that neuromarketing endeavours underlie ethical research codes to safeguard individuals (Dennett, 2010; Klemm, 2010; Brembs, 2011; Pereboom and Caruso, 2018).

The dilemma is outlined in Table 2. Situations A and B are the same in terms of the intention of the person, “*I’ll go to the show and buy X.”* The question is whether their purchase decision will be different, depending on situation A or B, in terms of the ultimately chosen product to purchase, number and types of additional products purchased (that were not part of the initial intention), etc.

**TABLE 2:** Illustration of two possible situations, outlining the dilemma of the effect of the presence of persuasive advertisement on ultimate purchase decisions.

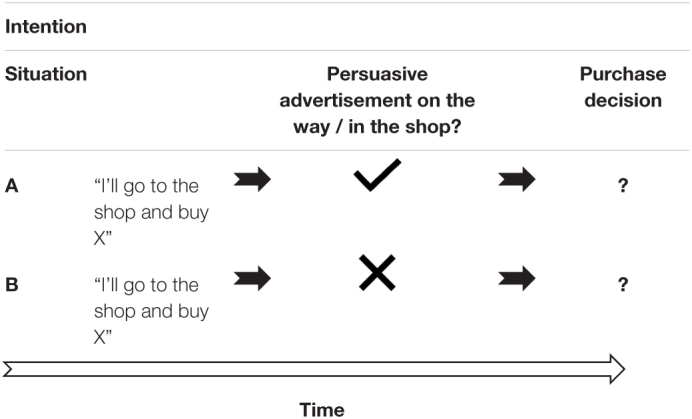

We can say that it is, *theoretically*, correct that people are free to choose, whether or not they buy a product. Freedom of choice is a basic human right, we are free; Article 1 of the The Universal Declaration of Human Rights (1948). However, considering what we know about human cognition today in the realm of consumer behaviour, we may doubt that our decisions are entirely “free” (e.g., Berthoud, 2007). This implies that we need (1) to safeguard individuals against manipulation that can lead to detrimental effects of the individual, societies, or the environment, (2) to regulate persuasive advertisement, and (3) to educate ourselves as consumers about how our brain can be manipulated during a purchase decision.

Empirical research shows that very often our choices and behaviours during purchasing decisions are, at least in part, determined by three main processes:

(1) *habit loops* (especially those related to basic need satisfaction);
(2) *elicitation of aesthetic emotions* (e.g., being moved, awed, attracted, made curious, impressed, etc.);
(3) *perceptual and cognitive biases* (e.g., number biases, and the distancing bias).

As we will see in the next sections, these three processes modulate our decisions. We will briefly review how (1) *habit loops* (Section “Habit Loops”), *aesthetic emotions* (Section “Move Me! Move Me to Act… Aesthetic Emotions”), and (3) *perceptual biases* (Section “Perceptual and Cognitive Biases”) condition our purchasing behaviour.

When is it ethical, and when is it not?

### Habit Loops

Is neuromarketing research simply aimed at benefitting the individual and society, by giving individuals and societies “*what they want and truly desire*” (Bernays, 1928/1955/2004; Dichter, 1960/2012/2017; Papakonstantinou, 2019)? In keeping with the idea set out above, that our decisions to purchase a product are not entirely free if persuasive advertising is used, the answer to this question is “no.” What we think we “need” and what the human body and brain actually need from a psychological and physiological health perspective, is not always aligned if persuasive ads target the habit loops of our brain.

Habit loops are learned cue-reactivities of our brain. Stimuli related to our basic needs (food, drink, shelter, sex, sleep; Maslow, 1943) trigger strong activations of the reward systems of the brain and strong subjective hedonic reactions (Kringelbach and Berridge, 2009, 2010b). Receiving “pleasure” causes us as individuals to develop a strong motivation to repeat the behaviour that led to this pleasure also in the future. As the contingency between stimulus and pleasure becomes stronger (“*if I do Z, I will feel pleasure*”), the organism is *learning*. This means that circuits in the brain become optimised into motivational loops that aid the execution of the behaviour in the future without much conscious effort. The basic principle at play here is known as operant conditioning in psychology, a basic principle of learning (Skinner, 1937, 1938, 1966, 1986). Thus, habits are learned behaviours (i.e., outcome/contingency-based learning) that require little conscious effort. This is basically a way to save energy resources for the brain (Knowlton et al., 1996; Shohamy et al., 2004; Bayley et al., 2005; Ashby et al., 2010).

The ability to develop habits is a very adaptive feature of the human brain, allowing optimisation of resources, and ultimately, optimisation of behaviour. It is helpful not having to plan out every sequence of behaviours that leads to a goal.

However, habits also leave a gap in our awareness. Therefore, it is important to supervise wisely which habits we develop. Considering that habits are behaviours that we *do*, that occur without much awareness, it is clear how habits can be dysfunctional for our health.

An example for a positive habit could be: **Cue** → “*going to bed*”; **behaviour associated with that cue** → “*brush your teeth*”; **reward associated with the behaviour:** “*fresh clean feeling in the mouth, no plaque.*”

An example of a habit that has negative consequences for our health could be: **Cue** → “*I feel nervous when I am with other people*”; **behaviour associated with that cue** → “*I grab a cigarette*”; **reward associated with the behaviour:** “*calm feeling, nervousness is gone*” (e.g., see Naqvi et al., 2007 for the social aspect of smoking addiction).

Some techniques from the (neuro-) marketing industry are directly aimed at bridging this habit-gap of our awareness to drive individuals toward purchase, irrespectively of any negative consequences for the individual, society or environment. The example of a habit with negative consequences above illustrates this. In this gap lies the challenge for the (neuro-) marketer of the future, to align their activity with current understandings of ethical research, and knowledge about the human brain. To put it slightly provocative: People don’t *need* the amounts of sugar that soft drinks contain, even if they *want* them. They also don’t *need* a smartphone of the newest generation whose expense sends their bank account into bankruptcy, even if they *want* it. And people also don’t *need* burgers that have a too low nutrient content for the amount of fat that they contain, even if they say that they really *want* that burger (Roos and Wndel, 2005; Zheng and Berthoud, 2007; Berridge et al., 2010).

Enjoying pleasure requires the right education (Christensen, 2017). The reason why people might be convinced that they *need* these things is because their brain has developed a habit and craves these items like a drug addict craves a drug (Köster, 2009). The same goes for other types of products, like downloadable music, games, or artworks. Even the pleasure that we feel from higher-order pleasures like music and arts, rely on the exact same neural substrates as drugs and addiction to drugs (Kringelbach and Berridge, 2009, 2010a,b). Hence, anything that leads to overuse of these products that can stimulate the reward circuitries and induce *craving* deprives us of our healthy decision-making behaviour. This can make our behaviour shift from being a conscious action to a bad habit, and from there to becoming a compulsion (Frijda, 1987; Delgado et al., 2005; Everitt and Robbins, 2005).

For example, high-calorie food cues in ads (designed with the help of neuromarketing efforts?) trigger our sugar habits and make us crave, just like a drug addict craves their drug. Research shows that if such potent habits are learned in early childhood, a high reactivity of the reward system to high-calorie food cues will never subside. This makes the development of obesity highly likely (Birch et al., 2007; Biro and Wien, 2010; Craigie et al., 2011; Movassagh et al., 2017).

Another aspect of a food/drink product that can induce such habits is another aspect of the product’s composition. The “bliss point” in food design refers to the perfect mix of sweet, fat and salty in a product, and is the result of a neuromarketing effort, aimed at finding the most hedonic mix of ingredients to make the product highly hedonic, provoke a strong pleasure response that will increase the motivation of the individual to ingest the juice again, each time a cue related to the juice appears. Such mixture, however, increases ghrelin (a so-called hunger hormone) concentration in blood, inducing hunger feelings in the individuals. In principle, this process is good, since it starts off digestion (saliva and gastrointestinal systems are set to be ready for digestion). However, these processes also initiate if the nutrient content of the food is zero, which is the case with junk food, that is deprived of nutrients (vitamins, minerals, fibres, etc.). The body, ready for digestion, will keep sending signals to the brain to ingest more, even if the caloric intake (calorie intake is not the same as nutrient intake) already surpasses healthy levels (Flood-Obbagy and Rolls, 2009; Halford and Harrold, 2012; Van Kleef et al., 2012; Moss, 2013), the result of this is overeating, and eventually, obesity.

Already in 1957, Packard expressed his concern in relation to the overuse of advertising, highlighting the risk of manipulating customers into over-consuming (Packard, 1957/2007), and puzzlingly, today, the food industry is still allowed to target children with persuasive advertising for products like sugary drinks.

To give one beat more detail of what we know today about sugary drinks’ effect on the body: From a nutritional point of view, there is absolutely no reason for a child to ingest sugar, considering current knowledge about the effects of sugar on the consumer’s brain and, subsequently, on their body, and then, subsequently, on the societies’ economic burden due to ill health and dysfunctional behavioural patterns. Besides, studies show that no level of processed fruit (juice, puree or juice with fibres) has the same effect on satiety as a real fruit (Wojcicki and Heyman, 2012a,b). In one study, participants were given a processed fruit serving or a real fruit to eat 15 minutes before a meal. After the meal, the group of people that had eaten the real fruit felt more satiated and felt fuller than any of the groups that had consumed processed options (Flood-Obbagy and Rolls, 2009). One study showed that serving fruit juice to children should potentially be questioned altogether, given the adverse health effects (Wojcicki and Heyman, 2012b).

This is a clear example where what people want and desire (more sweet juice), is not what they (their body) really need. And the reason why they “want” it, is the presence of cues related to the juice and the pleasure that is expected, which triggers the habit (behaviour) to ingest it. The same goes, for many other things that we may engage with, including products related to social media, films, music, gaming, pornography, etc. (Kalivas and Volkow, 2005; Grant et al., 2010; Freimuth et al., 2011; Gearhardt et al., 2011; Olsen, 2011; Alavi et al., 2012).

We may find the fine line that differentiates a healthy product or service from an unhealthy one by asking ourselves a question: Is the content of the product or service that the neuromarketer is hired to investigate junk or genuine? “Genuine” content is content that is ethical from the standpoint of today’s knowledge about the human brain, our societies, and our environment. This implies that the content, the advertising, the sales strategies etc., don’t hamper individuals’, societies’, and environmental health. By implication, the use of neuromarketing methods to boost sales with persuasive ads is not in itself bad. Let us end this section with some examples where knowledge about how the brain reacts to a conditioned cue has produced positive outcomes for individuals, as well as for industry.

First, persuasive advertising for toothpaste. In the 1950s, toothpaste manufacturers were not very successful in marketing their products, until they used knowledge about how attention to sensory cues can drive behavioural change, and even form “good habits.” “The sense of freshness with mint” became the cue that everyone could feel through brushing their teeth. The sensory cue that the persuasive ads proposed to get rid of it was: plaque (Fischman, 1997; Miskell, 2004; Aunger, 2007). Now, every time that consumers would identify “plaque” with their tongue on their teeth, they would know what to do: brush their teeth to get the taste of mint that is “so fresh” and “clean” (Hopkins, 1998; Hujoel, 2019). This boosted the sales numbers of the companies involved, increased individual health (because the use of toothpaste resulted in better dental health), and lessened the economic burden of the society due to ill dental health burden on the health services.

Second, resealable packages for a better self-regulation are another example (Plassmann et al., 2008; Reimann et al., 2010). Optimal food packaging strategies (with the right cues!) can help consumer self-regulation. One study found that if an energy-dense food product is offered in a resealable package, this helps consumers self-regulate their consumption and thus eat less palatable foods (De Bondt et al., 2017).

Third, reduce ad volume (Stallen et al., 2010). Ad volume is in itself a problem due to the sensory overstimulation that it entails for us in our everyday lives (Baumeister et al., 2008; Baumeister, 2014). Targetting segments of consumers more directly and selectively may help to reduce ad volume be the way forward more directly and selectively (Venkatraman et al., 2012), reducing ad volume. This is not an invitation to personalized ads regardless, but for ethically designed personalized ad procedures.

Fourth, neuroscientific techniques can also help gain deeper insights into the neurobiological mechanisms of compulsive purchasing, and then assist the development of awareness campaigns, e.g., in collaboration with policymakers and other stakeholders in the domain of health promotion (Black et al., 2000; Fortunato et al., 2014).

### Move Me! Move Me to Act… Aesthetic Emotions

Aesthetic emotions are another set of potent drivers of consumers’ choices. Aesthetic emotions are emotions that we feel in the everyday context, for example, in response to artworks, films, music, dances, architecture, nature scenes, etc., and also when faced with a product, service, person or idea that is being marketed. Aesthetic emotions include (but are not limited to), *being moved*, feeling *awe*, *fascination*, *surprize*, *suspense*, *elation*, *caring*, *tenderness*, *pity*, *shock*, *fear*, *guilt*, *shame*, *outrage*, *disgust*, etc, and they cause an affective reaction in us (Menninghaus et al., 2019). For empirically grounded theoretical stances about aesthetic emotions, see, e.g., the Multicomponent Model of Aesthetic Emotions (Menninghaus et al., 2019), or the Vienna Models of Aesthetic Appreciation (Leder et al., 2004; Pelowski and Akiba, 2011; Pelowski et al., 2016, 2017) and see also (Chatterjee, 2003, 2011) for the framework behind neuroaesthetics as a discipline. Physiological responses that occur when we have an aesthetic episode include, but are not limited to, unspecific activation patterns of the autonomic nervous system (measurable as changes in heart rate, galvanic skin response, pupillary responses, etc.), chills/goosebumps, tears, shivers (Pelowski and Akiba, 2011; Pelowski, 2015; Pelowski et al., 2016; Tinio and Gartus, 2018), that are likely related to the subjective experience of an emotional reaction to what is being perceived. Besides, aesthetic emotions may implicitly stir a call to action in us (Keltner and Haidt, 2003; Konecni, 2005; Markovic, 2010; Pelowski, 2015).

There are several aspects of a product or service that have the potential to elicit aesthetic emotions. As examples, take a look at the orangutan commercial and the MR W commercial, that induce us to reconsider the use of products that contain palm-oil, and to choose wind-energy if we can. Or, consider, this deeply *moving* commercial for a soap.

Anything that stirs our emotions can induce basic tendencies to approach or to withdraw in us – in terms of consumerism: to purchase or not. Emotions generally trigger two basic behavioural tendencies (pleasure and displeasure) that motivate individuals to either approach (and wish to move toward/possess), or withdraw from (and avoid/wish to discard) the stimulus or situation that is causing the affective reaction in us. Basic theories of motivation and emotion suggest that this affective reaction primes our behaviour (approach or avoid) (for a review, see Harmon-Jones et al., 2017). Such behavioural approach-avoid tendencies are also extensively studied with regards to food cues in the lab, as a measure of implicit bias toward certain foods, for instance, evidenced with push-pull (withdraw-approach) kind of experimental paradigms (Werthmann et al., 2014; Lender et al., 2018; Meule et al., 2019a,b). These findings provide insight into the biases in our choices that occur as a consequence of the emotional states that are evoked in us through a marketing effort (be it in product design or the promotion strategy).

Commercials that use strategies to trigger aesthetic emotions can change our behaviour for the better (“better” here meaning “enhancing” for individual, societal and environmental health). However, some other commercials give a different flavour, when the product itself is ethically questionable, for instance, due to its contents, or due to the way it is produced (respecting or not workers’ rights and environmental sustainability in the country and location of production).

Let us consider the following examples where consumers’ aesthetic emotions of curiosity, surprize, outrage, being shocked, and beauty are being used both to bond them to a product/brand but is at the same time used to reinforce racist or religious stereotypes:

For example, this clothing commercial, this beer commercial, and this detergent commercial. These campaigns thankfully stirred important social backlash. As did this other soap commercial for being racist (Simms, 2017; Wootson, 2017).

For the neuromarketer the basic question here is: the aesthetic emotions that I’m helping to trigger (e.g., awe, surprize, outrage, etc.), what are they for? Can they be harmful for consumers psychologically, or physically (Murray, 2013). It is important to ensure that Article 2 of the Charter of Human Rights is respected:

“*Everyone is entitled to all the rights and freedoms set forth in this Declaration, without distinction of any kind, such as race, colour, sex, language, religion, political or other opinion, national or social origin, property, birth or other status. [*…*]*.” (United Nations, 1948, Article 2)

We experience aesthetic emotions in response to stimuli that reach our senses (vision, hearing, touch, smell, taste). They have the potential to motivate us in one direction or another. We may feel change of mindset and want to improve something in our life after an aesthetic episode, like after a movie, a dance, a piece of music, or after having seen the commercial with Mister W. These emotions can, however, also be misused (like making people feel admiration for the ladies carrying the “torches of freedom” alluded to in Section “Examples of “Helping Consumers Get What They ‘Need’ “”. Spectators were induced to feel admiration and a drive to be like these “free” women with the Torches of Freedom, and therefore, to smoke).

### Perceptual and Cognitive Biases

In the hypothetical dilemma of the Orange Bubble Juice (in Section “Neuromarketing Present”), we mentioned the cognitive and perceptual biases that are being triggered wilfully, using the knowledge about biases of the human brain to condition consumer choices, especially, in the pricing and placement strategy of the product, including the use of ego-depletion strategies, number biases, etc.

Human choices are invariably infused by perceptual and cognitive biases. A long research tradition in psychology, and, more recently in cognitive neuroscience studies these irrational determinants of human choices (Tversky and Kahneman, 1981; Harmon-Jones and Mills, 1999; Kahneman and Tversky, 2000; De Martino et al., 2006; Santos and Rosati, 2015; Linares et al., 2019). Research in experimental psychology and cognitive neuroscience shows that repeatedly resisting temptations in an environment scattered with cues promising pleasure, where we must repeatedly regulate our affect and control our behavior, can deplete cognitive resources (Muraven and Baumeister, 2000; Saleh, 2012; Hirt et al., 2016; Martela et al., 2016), and affect the body’s basic energy supply (Vohs et al., 2005). When this resource is depleted, self-control may fail and decision making is impaired (Baumeister, 2002a,^2014^; Baumeister et al., 2008; Pocheptsova et al., 2009). Some research links ego deplesion to impulsive purchasing (Baumeister, 2002b; Sharma et al., 2010). Nevertheless, such an overcrowded visual field is approved, in every supermarket, everywhere in the world.

Another important cognitive bias is brought to our brain by celebrity endorsement. One study conducted in India showed that celebrity credibility has a significant impact on consumers’ attitudes toward the brand and advertisement, and on purchase intention (Singh and Banerjee, 2018). On the other hand, followers in social media may be connected emotionally to influencers which may increase the chance of behavioural inclination to accept the influencer’s endorsement (Bragg et al., 2016; Cuevas et al., 2020). Unfortunately, many such endorsements are for products that probably would not meet the criteria of being healthy (Zhou et al., 2019).

The question for the neuromarketer is always *why* they wish to trigger a perceptual or cognitive bias during the marketing process, and for *which* product, idea, person or service that they are being asked to assist a marketing endeavour. What is the final outcome and what it does to people ethical from the point of view of the Helsinki Declaration for Experimentation with Human Subjects?

## The Dilemma

It has been argued that if purchase behaviour of consumers in the market is left unprotected, it will ultimately become a catalyst for unscrupulous and unethical business practices (Titus and Bradford, 1996). This could lead even consumers that commit to ethical consumerism into a deep attitude-behaviour gap, where expressed attitudes (intention to be an ethical consumer) are not matched in behaviour (purchase decisions) (Chatzidakis et al., 2007). However, if policy makers propose regulations of the market, voices are very fast to say that this might put the economy at risk. Puzzlingly, by implication this means that the industry acknowledges that the strategies used are aimed at enticing consumers to over-consume. Detrimental effects on individual, societal and environmental health are discounted as “side-effects,” according to the utilitarian moral philosophy. The “Greater Good” here is the “health” of the economy, according to the consumerist economic model (Boström, 2005).

The type of threats that come from the industry toward policy makers were exemplified in July 2020 where the United Kingdom was to introduce a ban on sugary food ads before 9PM because a clear positive relationship between obesity and Covid-19 had been found. The proposal to ban certain ads before 9PM came from the United Kingdom Prime Minister.

Economists were fast to condem this intention to ban such ads as “dangerous.” One article warned that the predicted cost to the United Kingdom’s economy of the ad ban would be around $1.3 billion, and that this ban would probably lead to a raise in prices for consumers (Morales and Swint, 2020). It was summarised by the chief operating officer at the Food and Drink Federation, as *“[*…*] new restrictions on promoting and advertising everyday food and drink will increase the price of food, reduce consumer choice and threaten jobs across the U.K.*”. A different outlet read “*The government’s new obesity strategy for England will raise prices, reduce consumer choice, threaten jobs and stifle innovation. And all to save 17 calories a day*” (Morrison, 2020); using “ridiculing” to take credibility from the proposal (i.e., “17” calories doesn’t seem much making the possible gains of the proposal seem insignificant, on the expense of a great cost).

Let us consider this dilemma that the Prime Minister now faced through the lens of research on moral judgement (for a review, see Christensen and Gomila, 2012). The prime minister stood before this choice:

(A) ban ads before 9pm and risk jobs,

*vs*.

(B) allow the ads before 9PM and risk an obesity pandemic on top of the Covid pandemic.

**FIGURE 4:**
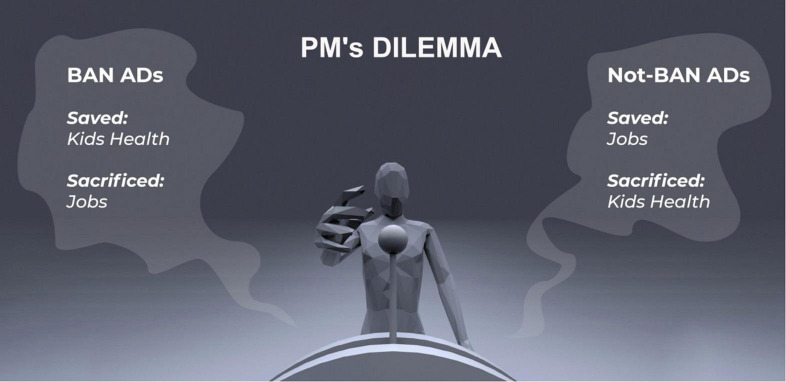
The moral dilemma faced by the UK Prime Minister. Observe the two possible judgements about this dilemma: we can choose to refrain from acting (and cause harm by omission we don’t act, or we can choose to act (and cause harm by commission this will save people (because they don’t get obese in the first place), but harm people that will lose their jobs until the economy has restructured itself. Designed by all authors. Illustration by Sina HN Yazdi.

In moral judgement research, a dilemma is a hypothetical situation where two different chains of events are possible. Each chain of events leads to some type of harm. These are different types of harm, but harm nonetheless. Research participants are then asked to, hypothetically, *choose* which of the proposed chains of events they prefer. For example: do you pull a switch to redirect a trolley so that it changes its course and kills one person instead of five people that would die if you don’t intervene? (Foot, 1978; Thompson et al., 1981; Greene et al., 2001). Similarly, the Prime Minister faced a dilemma about who to save (children or jobs?), and who to sacrifice (children or jobs?). See Figure 4.

In addition, another ingredient that we know from moral judgement research was present in the prime minister’s dilemma: our own relationship to the saved or to be “sacrificed” individuals, is a factor that has been called “Benefit Recipient” in moral judgement research (Christensen et al., 2014). The background at the time was that the Prime Minister had just recovered from a serious Covid-19 episode, that likely had been exacerbated by his own high BMI, and he had become a father to a baby-son. Thus, his choice was suddenly not only a dilemma between jobs and health, but also a very personally relevant moral dilemma. The dilemma had now become a type of dilemma, where the outcome, if the agent choose to act (i.e., here: ban ads for unhealthy foods before 9PM), would be a “self-beneficial” outcome, because it would be benefical to the agent themselves or their kin. The individuals to save, by imposing an ad-ban of this kind, are in this case himself and his baby-son. As evidenced by the research on moral judgement, it is always easier to consent harm (in this case to “the economy”), if the harm produced is “self-beneficial,” i.e., relevant to our own benefit.

At present, the above example of a successful ban for palatable drinks ads is rather unusual. The most common situation is that there are stark warnings from potent stakeholders that “overregulating” food, drink and cigarette markets would be turning countries into “nanny-states” that will feel patronising for citizens (Rouch et al., 2010; Wheeler, 2018; Nanny State Index, 2019). For sure, European countries like the United Kingdom are considered among the most patronising in this respect, and European countries like Germany among the least so (Austin, 2016; Nanny State Index, 2019). Generally, citizens dislike a state that aims to “protect” them from themselves’ (Kwon et al., 2019), for instance, in terms of food policies to promote healthy diets (Kwon et al., 2019), and it is true that more regulation isn’t always the best solution (Peters et al., 2013).

However, from a different point of view, “*The nanny state critique is ultimately a call for the state to be agnostic about the health of citizens, allowing market forces to dominate*” (Magnusson, 2015, p. 1). Besides, as reviewed above, also research in cognitive neuroscience shows that the story isn’t as easy as that, that we are not as free as we think we are, when we choose to purchase something that is detrimental to our health. Therefore, adopting a view that sees “*legislation* [as a way that] *brings about changes that individuals on their own cannot, and sets new standards for the public good. Rather than condemning such activity as ‘nanny statist’, it might be more appropriate to view it as a form of ‘stewardship’*” (Jochelson, 2006, p. 1), which seems more adequate. Such legislation might be informed, among other factors, by what we know about the human psyche and brain at present from psychology and neuroscience.

On a very basic level, legislating neuromarketing research practice following the principles set forth in the Helsinki Declaration for Experimentation with Human Subjects would be a first important step. Then the point would be about identifying, via democratic processes, what basic liberties a society can agree on with regards to (neuro-)marketing endeavours, and which then should be protected by law. This is a different form of freedom than leaving citizens to their own fate, full well *knowing* that this will contribute, for instance, to the global social and economic burden of poor health (Pettit, 2015; Kwon et al., 2019).

## Final Considerations

On the basis of our non-exhaustive analysis of available literature in this domain throughout this article, we propose several take-home messages:

(1) ***Scientists/academics at universities and research institutions working with industry:***
• Read the Helsinki Declaration of Helsinki for Medical Research Involving Human Subjects, especially regarding the points (i) use of deception, (ii) how to safeguard individuals’ dignity, (iii) adequate use of methods, and (iv) how to safeguard effects on the environment.
• Perform a proper risk assessment prior to any research activity (as is costumery within academic research), specifically regarding whether the evidence that would be gathered with this research could be misused to exploit individual, societal and the environmental health to favour financial gains of a small number of individuals. And, work minimise any such risk.
(2) ***Companies, classical marketers, producers of ads, artists working in the marketing business, etc.*:**
• Consult professionals that have a scientific background and who are trained in applying ethical research practices.
• Collaborate only with adequately trained scientists who are able to apply the right methods, and at the same time can contribute to elaborate cost-benefit plans as well as risk assessments regarding potential risks to individuals, society and environment, while keeping the potential benefits for the company in mind.
(3) ***Policy makers, individuals working in NGOs, etc*:**
• Evaluate the potential impact of certain neuromarketing practices for individual, societal and the environmental health and wellbeing, with the help of adequately trained scientists.
• Undertake risk assessments to inform whether regulatory endeavours are needed to safeguard individuals, the society and/or the environmental from some neuromarketing practices.
• Contribute to the discussion about *whether* and *which* risk-signalling endeavours might be useful on packaging etc., to inform consumers, and societies of potential risk (e.g., as proposed as a “disclaimer” in our provocative Hypothetical Orange Juice Dilemma example).
(4) ***Individuals*:**
• Educate oneself regarding the effects of persuasive advertising on our body and brain, and ultimately on our decision making in the consumer choice domain.
• Avoid exposing ourselves to potential manipulation of persuasive ads (e.g., switch off TV during ad blocks, instal ad blocker in computer browser, etc.).
• Educate children as much about the risks of too much fat and sugar intake, as of the hazards of succumbing to a persuasive advertisement.

### Conclusions

The Helsinki Declaration (1964) is inspired by the The Universal Declaration of Human Rights (1948). It was brought forward after the turmoil of the Second World War, to prevent that, ever again, research should be carried out that fails to safeguard individual, societal and environmental health and wellbeing. It has been endorsed additionally by several international directives since then (US Department of Health and Human Services, 1979; Iphofen, 2009; European Commission, 2018), and it has been clearly stated that these apply as much to medical research, as to social and behavioural research endeavours. This means, these guidelines for ethical research also apply, in their entirety, to the domain of Neuromarketing research.

We have outlined that both individuals as consumers, and policy makers assuming stewardship, can assist the neuromarketing researcher to move toward an ethical research practice in this domain.

### Limitations

Individuals (consumers), the neuromarketer and the steward face important obstacles. The consumer, due to the biases of our human brain, the neuromarketer due to pressures from industry and the need to earn a living, and the policy maker, due to the warnings and threats from the industry of job losses and economic hardship if the marketing effort is regulated.

### Outlook

The Neuromarketing Science and Business Association (NMSBA) has provided a Code of Ethics, that members can sign up to voluntarily. This code takes into account precisely the important ethical guidelines that should govern any research with human subjects. Such self-regulatory efforts should be promoted extensively. The most important step for neuromarketers of the future, as much as for companies working in the marketing business in general, is to familiarize themselves with the available ethical guidelines that regulate research with human participants in general, and then apply these to the (neuro)marketing domain. The Code of Ethics of the NMSBA provides a useful starting point.

## Author Contributions

JC: conceptualisation, literature search, main responsible for providing drafts and revisions of the manuscript. FF: conceptualisation, literature search, provided revisions of drafts, and lead discussion rounds. MV: literature search, provided critical revisions of manuscript drafts, content check, and reference management. SY: conceptualisation of summary figures, critical revisions of manuscript drafts, and content check. All authors contributed to the article and approved the submitted version.

## Conflict of Interest

FF and SY were employed by 3Fish Corporate Filmmaking, Tehran, Iran. The remaining authors declare that the research was conducted in the absence of any commercial or financial relationships that could be construed as a potential conflict of interest.

## Publisher’s Note

All claims expressed in this article are solely those of the authors and do not necessarily represent those of their affiliated organizations, or those of the publisher, the editors and the reviewers. Any product that may be evaluated in this article, or claim that may be made by its manufacturer, is not guaranteed or endorsed by the publisher.
